# Supplementary material for: Linking patient-reported oral and general health-related quality of life
Source: PeerJ. 2024 May 28;12:e17440. doi: 10.7717/peerj.17440 (PMC11141547; doi:10.7717/peerj.17440)
Supplement: Supplemental Information 2 [file peerj-12-17440-s002.docx]

STROBE Statement—checklist of items that should be included in reports of observational studies

|  | Item No. | Recommendation | Page  No. | Relevant text from manuscript | |
| --- | --- | --- | --- | --- | --- |
| **Title and abstract** | 1 | (*a*) Indicate the study’s design with a commonly used term in the title or the abstract | 2 | “**Methods:** This cross-sectional study used a convenience…” | |
|  |  | (*b*) Provide in the abstract an informative and balanced summary of what was done and what was found | 2 | This is covered in the **Methods** and **Results** section of abstract. | |
| Introduction | | | |  |  |
| Background/rationale | 2 | Explain the scientific background and rationale for the investigation being reported | 3-4 | **Introduction** | |
| Objectives | 3 | State specific objectives, including any prespecified hypotheses | 4 | **Introduction**: “This study aimed to determine the OHRQoL-HRQoL relationship over a longer period, employing a 12-month recall period, the OHIP-5, and the 10-item PROMIS 1.2 – Global Health Instrument within an adult community sample to provide a deeper understanding of the OHRQoL-HRQoL relationship within an adult community sample... By using this practical approach, we seek to contribute to a broader awareness of the pivotal role oral health plays in the holistic well-being of individuals, ultimately fostering improved healthcare strategies.” | |
| Methods | | | |  |  |
| Study design | 4 | Present key elements of study design early in the paper | 4-5 | **Participants and study design** paragraph | |
| Setting | 5 | Describe the setting, locations, and relevant dates, including periods of recruitment, exposure, follow-up, and data collection | 4-5 | **Participants and study design** paragraph | |
| Participants | 6 | (*a*) *Cohort study*—Give the eligibility criteria, and the sources and methods of selection of participants. Describe methods of follow-up  *Case-control study*—Give the eligibility criteria, and the sources and methods of case ascertainment and control selection. Give the rationale for the choice of cases and controls  *Cross-sectional study*—Give the eligibility criteria, and the sources and methods of selection of participants | 4-5 | n/a  n/a  “…English-speaking adults aged 18 years or older were conveniently sampled for participation. Recruitment took place in-person at two prominent Minnesota county fairs and at the Minnesota State Fair… Interested fairgoers voluntarily approached the study booth to express their willingness to participate. Following a comprehensive explanation of the study, participants underwent the informed consent process, written consent, enrollment, and subsequently completed the study activities.” | |
|  |  | (*b*) *Cohort study*—For matched studies, give matching criteria and number of exposed and unexposed  *Case-control study*—For matched studies, give matching criteria and the number of controls per case | n/a  n/a | n/a  n/a | |
| Variables | 7 | Clearly define all outcomes, exposures, predictors, potential confounders, and effect modifiers. Give diagnostic criteria, if applicable | 3  6  6  8 | OHRQoL and HRQoL Defined: “Oral diseases impact patients in one or more of the four OHRQoL dimensions; Oral Function, Orofacial Pain, Orofacial Appearance, and Psychosocial Impact which represent the elemental building blocks of OHRQoL.(John et al., 2014a,b) The same applies for diseases which impact HRQoL in its two dimensions, Physical Health and Mental Health.”  Potential confounders:  “…after adjusting for the influence of gender, age, and teeth count.”  “Then, the influence of gender, age, and teeth count was investigated by adjusting the model for these covariates.”  “Adjustment for other factors, such as depression…” | |
| Data sources/ measurement | 8* | For each variable of interest, give sources of data and details of methods of assessment (measurement). Describe comparability of assessment methods if there is more than one group | 5  5  5-6 | See “**Physical oral health measurement**” for details of physical assessment.  See “**Oral health-related quality of life (OHRQoL) measurement**” for details of OHRQoL assessment using OHIP-5  See “**Health-related quality of life (HRQoL) measurement**” for details of HRQoL assessment using PROMIS v.1.2 General Health. | |
| Bias | 9 | Describe any efforts to address potential sources of bias | 9-10 | “It is important to acknowledge a limitation related to the voluntary nature of participation in our study. It is possible that individuals with better oral and/or general health were more inclined to participate. This self-selection bias may introduce a potential source of selection bias in our results, as those who chose to take part may not be entirely representative of the broader population.” | |
| Study size | 10 | Explain how the study size was arrived at | 5 | No sample size calculation was conducted due to the unique mode of recruitment which depended on attendance at county and State fairs. The goal was to sample as many participants as possible. We do say that “Interested fairgoers voluntarily approached the study booth to express their willingness to participate.” | |
| Quantitative variables | 11 | Explain how quantitative variables were handled in the analyses. If applicable, describe which groupings were chosen and why | 6 | Explanation of how quantitative variables were handled can be found in “**Data Analysis**.”  Further details in Figures 1 and 2. |  |
| Statistical methods | 12 | (*a*) Describe all statistical methods, including those used to control for confounding | 6 | Statistical methods including use of bivariable correlation and structural equation modelling described in “**Data Analysis.**”  Further details in Figures 1 and 2. |  |
|  |  | (*b*) Describe any methods used to examine subgroups and interactions | n/a | n/a |  |
|  |  | (*c*) Explain how missing data were addressed | 7 | “Participants were excluded if they had missing data on any of the variables of interest (OHIP-5 summary score, mental HRQoL, physical HRQoL, gender, age, and total number of teeth) for a sample size of N=618. This data loss comprised of less than 3% of our initial sample of 635.” |  |
|  |  | (*d*) *Cohort study*—If applicable, explain how loss to follow-up was addressed  *Case-control study*—If applicable, explain how matching of cases and controls was addressed  *Cross-sectional study*—If applicable, describe analytical methods taking account of sampling strategy | n/a  n/a  n/a | n/a  n/a  n/a |  |
|  |  | (*e*) Describe any sensitivity analyses | 6 | “A series of SEMs were generated to investigate the relationship between OHRQoL and HRQoL. We fit four models to these data by treating polytomous OHRQoL and HRQoL items as categorical, using unweighted least squares estimation….The primary model (Model 1) only considered OHRQoL and HRQoL whereas the sensitivity model (Model 2) evaluated the residual correlation between OHRQoL and HRQoL after adjusting for the influence of gender, age, and teeth count (Fig. 1)…Again, we first evaluated only OHRQoL and HRQoL without considering additional variables (Model 3). Then, the influence of gender, age, and teeth count was investigated by adjusting the model for these covariates (Model 4).” |  |
| Results | | | | |  |
| Participants | 13* | (a) Report numbers of individuals at each stage of study—eg numbers potentially eligible, examined for eligibility, confirmed eligible, included in the study, completing follow-up, and analysed | 7 | “A total of 635 were conveniently sampled. Participants were excluded if they had missing data on any of the variables of interest (OHIP-5 summary score, mental HRQoL, physical HRQoL, gender, age, and total number of teeth) for a sample size of N=618. This data loss comprised of less than 3% of our initial sample of 635. Those who reported a gender other than male or female, or preferred not to report their gender were also excluded from the analysis due to very small numbers in these individual categories. Thus, our final sample size was N=607.” |  |
|  |  | (b) Give reasons for non-participation at each stage | 5  7 | “Interested fairgoers voluntarily approached the study booth to express their willingness to participate. Following a comprehensive explanation of the study, participants underwent the informed consent process, written consent, enrollment, and subsequently completed the study activities. Ethical oversight was ensured by the University of Minnesota (UMN) Institutional Review Board (IRB), which granted approval for all study procedures under the study ID: STUDY00016028. Both OHRQoL and HRQoL instruments were self-administered by the participants”  “Participants were excluded if they had missing data on any of the variables of interest (OHIP-5 summary score, mental HRQoL, physical HRQoL, gender, age, and total number of teeth) for a sample size of N=618. This data loss comprised of less than 3% of our initial sample of 635. Those who reported a gender other than male or female, or preferred not to report their gender were also excluded from the analysis due to very small numbers in these individual categories. Thus, our for a final sample size of N=607” |  |
|  |  | (c) Consider use of a flow diagram | n/a | Due to the simplicity of the study design, authors considered, and decided against a flow diagram. |  |
| Descriptive data | 14* | (a) Give characteristics of study participants (eg demographic, clinical, social) and information on exposures and potential confounders | 7 | “The mean age of participants was 43.7 (SD 17.6), with 68% being female (Table 1). The majority of participants were white (90.8%), and not Hispanic or Latino (89.1%). The average OHIP=5 summary score of 17.1 (SD 3.3), out of a possible 20, indicating that this population did not suffer substantially from oral health impacts as a higher score indicates better OHRQoL. Physical and mental health dimensions scores had means of 16.2 (SD 2.3) and 15.8 (SD 3.1), respectively.”  More study participant characteristics are included in Table 1. |  |
|  |  | (b) Indicate number of participants with missing data for each variable of interest | 7 | “Participants were excluded if they had missing data on any of the variables of interest ( OHIP-5 summary score, mental HRQoL, physical HRQoL, gender, age, and total number of teeth) for a sample size of N=618. This data loss comprised of less than 3% of our initial sample of 635.” |  |
|  |  | (c) *Cohort study*—Summarise follow-up time (eg, average and total amount) | n/a | n/a |  |
| Outcome data | 15* | *Cohort study*—Report numbers of outcome events or summary measures over time | n/a | n/a |  |
|  |  | *Case-control study—*Report numbers in each exposure category, or summary measures of exposure | n/a | n/a |  |
|  |  | *Cross-sectional study—*Report numbers of outcome events or summary measures | 7  7 | “The average OHIP=5 summary score of 17.1 (SD 3.3), out of a possible 20, indicating that this population did not suffer substantially from oral health impacts as a higher score indicates better OHRQoL.”  “Physical and mental health dimensions scores had means of 16.2 (SD 2.3) and 15.8 (SD 3.1), respectively.” |  |
| Main results | 16 | (*a*) Give unadjusted estimates and, if applicable, confounder-adjusted estimates and their precision (eg, 95% confidence interval). Make clear which confounders were adjusted for and why they were included | 7  7  7  8 | Unadjusted estimates:   - “An assessment of the relationship between physical HRQoL and OHRQoL yielded a correlation of r=0.39 (95% CI: 0.32, 0.46). For the mental HRQoL and OHRQoL relationship, a slightly lower correlation of r=0.32 (95% CI: 0.25, 0.39) was observed.” - Like the bivariable correlations, the SEM models also showed that OHRQoL correlated slightly higher with physical HRQoL than with mental HRQoL, r=0.55 and r=0.43, respectively (Table 2). The 95% confidence interval widths of 0.09 and smaller indicated sufficient precision around point estimates.”   Confounder-adjusted:   - Adjustment for gender, age, and the number of teeth did not notably change the observed correlation estimates. - “Adjustment for other factors, such as depression, should lower the HRQoL-OHRQoL correlation; however, results were mixed. While the present study found a minimal decrease when depression was accounted for…” |  |
|  |  | (*b*) Report category boundaries when continuous variables were categorized | n/a | n/a |  |
|  |  | (*c*) If relevant, consider translating estimates of relative risk into absolute risk for a meaningful time period | n/a | n/a |  |
| Other analyses | 17 | Report other analyses done—eg analyses of subgroups and interactions, and sensitivity analyses | n/a | n/a |  |
| Discussion | | | | |  |
| Key results | 18 | Summarise key results with reference to study objectives | 8 | “This study showed the magnitude of the association between OHRQoL and HRQoL to be r=0.52 (95% CI: 0.50, 0.55), indicating that the two constructs shared 27% of their information. According to guidelines for the interpretation of a correlation’s magnitude. (Cohen, 1988; Cohen, 1992) This study provides evidence of a “large” correlation between OHRQoL and HRQoL in a USA-based general adult population.” |  |
| Limitations | 19 | Discuss limitations of the study, taking into account sources of potential bias or imprecision. Discuss both direction and magnitude of any potential bias | 9 | “It is important to acknowledge a limitation related to the voluntary nature of participation in our study. It is possible that individuals with better oral and/or general health were more inclined to participate. This self-selection bias may introduce a potential source of selection bias in our results, as those who chose to take part may not be entirely representative of the broader population.” |  |
| Interpretation | 20 | Give a cautious overall interpretation of results considering objectives, limitations, multiplicity of analyses, results from similar studies, and other relevant evidence | 9  9 | “With fewer OHIP variables being present in the OHIP-5, attenuation of the correlation, and greater error, was expected in comparison to the studies that used the OHIP-49 for OHRQoL measurement.”  “Our results fit well with literature findings even if the constructs HRQoL and OHRQoL can both be captured with a number of methodological options which include a variety of available instruments, version, recall periods to select from, and analytic approaches.” |  |
| Generalisability | 21 | Discuss the generalisability (external validity) of the study results | 4  8  9-10 | “Finally, while chance, i.e., sampling variability, adds to the complexity of correlation findings, the OHRQoL-HRQoL correlation can be truly different in varying populations.”  “This study provides evidence of a “large” correlation between OHRQoL and HRQoL in a USA-based general adult population.”  “It is important to acknowledge a limitation related to the voluntary nature of participation in our study. It is possible that individuals with better oral and/or general health were more inclined to participate. This self-selection bias may introduce a potential source of selection bias in our results, as those who chose to take part may not be entirely representative of the broader population.” |  |
| Other information |  | | | |  |
| Funding | 22 | Give the source of funding and the role of the funders for the present study and, if applicable, for the original study on which the present article is based | 10 | “This research was supported by the National Institutes of Health’s National Center for Advancing Translational Sciences, grant UL1TR002494. The content is solely the responsibility of the authors and does not necessarily represent the official views of the National Institutes of Health’s National Center for Advancing Translational Sciences.” |  |

*Give information separately for cases and controls in case-control studies and, if applicable, for exposed and unexposed groups in cohort and cross-sectional studies.

**Note:** An Explanation and Elaboration article discusses each checklist item and gives methodological background and published examples of transparent reporting. The STROBE checklist is best used in conjunction with this article (freely available on the Web sites of PLoS Medicine at http://www.plosmedicine.org/, Annals of Internal Medicine at http://www.annals.org/, and Epidemiology at http://www.epidem.com/). Information on the STROBE Initiative is available at www.strobe-statement.org.
